# Supplementary material for: Impaired immune surveillance accelerates accumulation of senescent cells and aging
Source: Nat Commun. 2018 Dec 21;9:5435. doi: 10.1038/s41467-018-07825-3 (PMC6303397; doi:10.1038/s41467-018-07825-3)
Supplement: Supplementary file 1 — Supplementary Information [file 41467_2018_7825_MOESM1_ESM.pdf]

SUPPLEMENTARY INFORMATION

Impaired immune surveillance accelerates accumulation of senescent cells and aging

Ovadya et al.

Content

Supplementary Figure 1 Impaired cell cytotoxicity in immune cells from old animals

Supplementary Figure 2 Clearance of senescent cells is impaired in *Prf1*$^{-/-}$ mice

Supplementary Figure 3 *Prf1*$^{-/-}$ mice develop chronic systemic and local inflammation

Supplementary Figure 4 *Prf1*$^{-/-}$ mice exhibit compromised organ function and impaired tissue integrity

Supplementary Figure 5 *Prf1*$^{-/-}$ mice exhibit reduced fitness and early onset of age-related pathologies

Supplementary Figure 6 Treatment with ABT-737 counteracts accelerated aging process of *Prf1*$^{-/-}$ male mice

Supplementary Figure 7 Treatment with ABT-737 increases median lifespan of progeroid mice

Supplementary Figure 8 Analysis of flow-cytometry and Imagestream X data

Supplementary methods

In vivo toxicity assay

To elicit specific $CD8^+$ T cells, mice were DNA vaccinated with the mammalian expression vector pCI encoding the Hepatitis B Core Protein. 12 days after immunization, mice were adoptively transferred with target cells. As control also one non-immunized (naïve) animal per group was transferred. As target cells CFSE labeled splenocytes (isolated from non-treated young animals) were used. Splenocytes were either surface coated with the non-relevant peptide "Ova" derived from Ovalbumin (labeled as CFSE 'low') or were coated with the specific Hepatitis B Core derived peptide "Core" (labeled as CFSE 'high'). CFSE 'high' and CFSE 'low' cells were mixed at a ration of 1:1 and were transferred into the immunized young and old mice, as well as into the naïve control animals. 5 hours after the adoptive transfer, recipients were sacrificed and spleens were harvested. Spleens were analyzed for CFSE-labeled cells using flow cytometry and frequency of CFSE 'high' and CFSE 'low' cells was determined. Specific killing was then calculated according to this formula:

$$\%\, specific\ killing = \left[1 - \left(\frac{\frac{Percentage\ CFSE\ 'high'\ immunized\ recipient}{Percentage\ CFSE\ 'low'\ immunized\ recipient}}{\frac{Percentage\ CFSE\ 'high'\ naive\ recipient}{Percentage\ CFSE\ 'low'\ naive\ recipient}}\right)\right] \cdot 100$$

Flow cytometry

Characterization of immune subsets in tissues was preformed by flow cytometry 1. Briefly, liver, pancreas, and lung from 2-month-old Prf1-/- and WT female mice were perfused, harvested, and dissociated to single-cell suspensions. Cells were stained with Violet Live/Dead viability dye (Life technologies) according to manufacturer's instructions, incubated with blocking solution (5% mouse serum, 5% rat serum, and 1% FcBlock (eBiosciences) in PBS), and stained with a standard pannel of immunophenotyping antibodies. The antibodies used in the pannel are Pacific Blue anti-CD45 (#103126, Biolegend), APC anti-Ly6G (#127614, Biolegend), PerCP-cy5.5 anti-CD11b (#101228, Biolegend), FITC anti-CD11c (#117306, Biolegend), APC/Cy7 anti-IA/IE (#107628, Biolegend), Brilliant Violet 605 anti-CD24 (#311124, Biolegend), PE and anti-CD64 (#139304, Biolegend) for 30 minutes in room temperature. The dilution of these antibodies was 1:100 for the studies. Cells were washed and fixed with 0.4% paraformaldehyde in PBS. Cells were analyzed in a SORP-LSRII instrument (BD Biosciences) and FlowJo v10 software. The cell gating strategy is shown in the **Supplementary Figure 8b**.

PCR Primers

The following primers were used: *p16* forward 5‘-CGTACCCCGATTCAGGTG-3‘, reverse 5‘-ACCAGCGTGTCCAGGAAG-3; *Il6* forward 5‘-AGACAAAGCCAGAGTCCTTC-3‘, reverse 5‘-TTCTGTGACTCCAGCTTATC-3; *KC* forward 5‘-AAGAATGGTCGCGAGGCTTG -3‘, reverse 5‘-TGCCATCAGAGCAGTCTGTC -3; *Il1a* forward 5‘-TCAACCAAACTATATATATCAGGATGTGG-3‘, reverse 5‘-CGAGTAGGCATACATGTCAAATTTTAC-3; *Il1b* forward 5‘-TGTAATGAAAGACGGCACACC-3‘, reverse 5‘-TCTTCTTTGGGTATTGCTTGG-3; *JE* forward 5‘-GCTCAGCCAGATGCAGTTAA-3‘, reverse 5‘-TCTTGAGCTTGGTGACAAAAACT-3; *GAPDH* forward 5‘-GACAGTCAGCCGCATCTTC-3‘, reverse 5‘-CGTTGACTCCGACCTTCAC -3.

RNA-seq data analysis

Analysis of RNA-seq data: Raw reads were mapped to the genome (NCBI37/mm9) using hisat (version 0.1.6). Analysis was carried using edgeR Bioconductor R-package version 3.18.1 (http://bioinf.wehi.edu.au/edgeR [2-4]). The analysis is based on a negative binomial model that uses over-dispersion estimates to account for biological variability. We filtered lowly expressed genes, used TMM library normalization, followed by tagwise dispersion estimate. We used exact test (“classic approach”) to perform pair-wise comparisons for detection of differentially expressed genes between experimental groups containing at least 3 biological replicates. Genes with a Benjamini–Hochberg false discovery adjusted P value $< 5e^{-2}$ were reported to be differentially expressed (no effect size cut-off was set). To identify pathways and biological functions, enrichment analysis of Gene Ontology (GO) was carried. The analysis was applied to two sets of differential gene, obtained by considering all tissues together and clustering by k-means into two main clusters (increasing and decreasing). GO annotations were cropped using GOSemSim [5], semantic similarity calculator, and subsequent hierarchical clustering (R base package), and for each cluster top scoring GO terms were selected. For total SASP scores, statistical significance was calculated using paired t-test for genes log ratios compared to young (kidney: t = 3.6, df = 24, p-value = $1.1e^{-3}$, 95 percent confidence interval: 0.16, 0.57; liver: t = 2.4, df = 19, p-value = $2.6e^{-2}$, 95 percent confidence interval: $9.1e^{-2}$ 1.2; lung: t = 0.64, df = 32, p-value = 0.52, 95 percent confidence interval: -0.18 0.35; skin: t = 3.9, df = 33, p-value = $4.1e^{-4}$, 95 percent confidence interval: 0.28 0.90). Linear regression statistics were calculated using t-statistic (Error = $4.5e^{-3}$, df = 7978). Pink spots represents the genes in the pink intersection area excluding 104 genes that only overlap between tissues. Spearman correlation analysis statistics were calculated using algorithm AS 89.

Supplementary figures

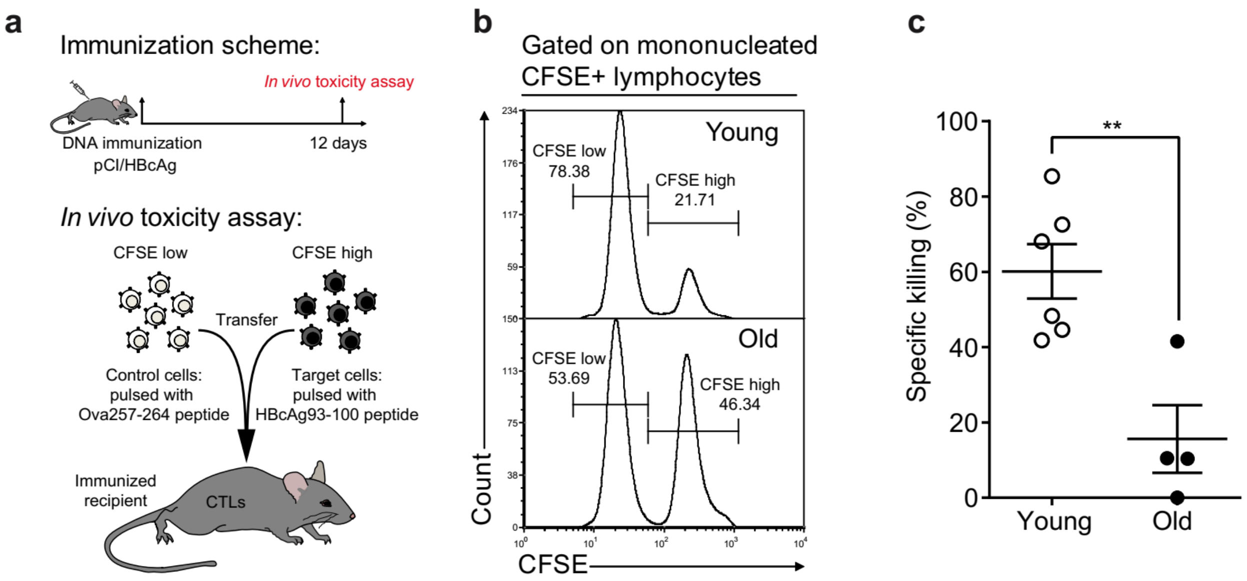

Supplementary Figure 1 Impaired cell cytotoxicity in immune cells from old animals. (**a**) Scheme describing the experimental setting which includes immunization of young and old mice against pCI/HBcAg, followed by *in vivo* toxicity assay. (**b**) Flow-cytometry analysis of mono-nucleated CFSE+ lymphocytes 5 hours post adoptive transfer to immunized young and old mice. Shown are representative histograms of the underlying analysis. (**c**) Quantification of specific killing of target cells in young and old mice, 5 hours post adoptive transfer. (** $P < 0.01$).

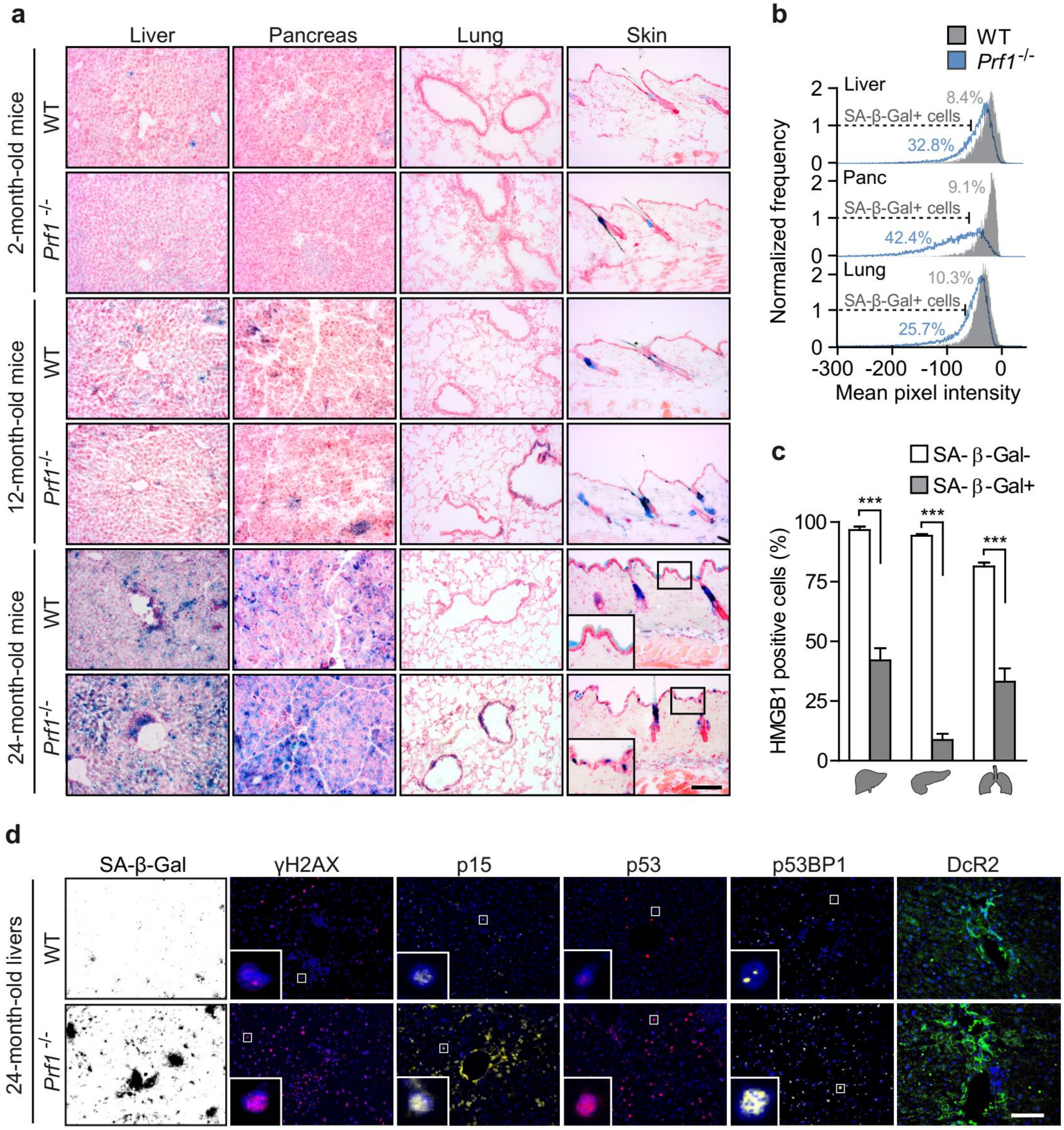

Supplementary Figure 2 Clearance of senescent cells is impaired in *Prf1*$^{-/-}$ mice. (**a**) SA-β-Gal activity representative frozen sections of liver, pancreas, lung, and skin from 2, 12, and 24 months old *Prf1*$^{-/-}$ and WT female mice. Scale bar, 100 µm. (**b**) ImageStreamX-derived histograms of SA-β-Gal activity in cells from liver, pancreas and lung. (**c**) ImageStreamX analysis for percentage of nuclear HMGB1+ cells in SA-β-Gal positive and negative populations from liver, pancreas and lung. Values are means ± SD, *n* = 5. (**d**)

Representative images of immunofluorescence staining of livers from *Prf1*$^{-/-}$ and WT mice with γH2AX, p15, p53, p53BP1, and DcR2 and for SA-β-Gal activity. Scale bar, 100 µm. (*** $P < 0.001$).

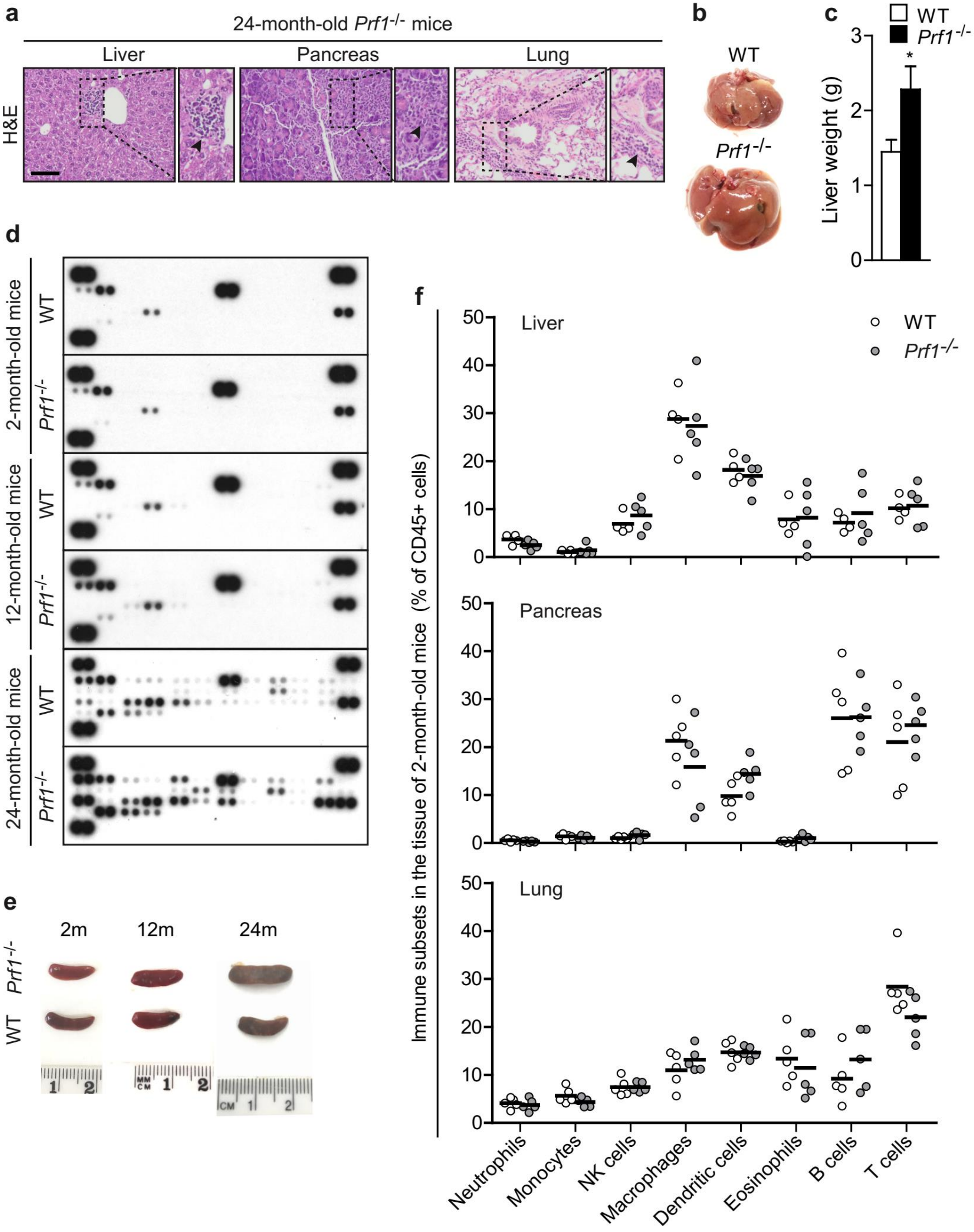

Supplementary Figure 3 *Prf1*$^{-/-}$ mice develop chronic systemic and local inflammation. (**a**) Representative images of H&E-stained sections of liver, pancreas, and lung from 24 months old *Prf1*$^{-/-}$ and WT female mice. Scale bar, 100 µm. Arrows point foci of immune infiltrates in the designated tissues. (**b**) Representative photos of livers from old *Prf1*$^{-/-}$ and WT mice. (**c**) Liver weights of the *Prf1*$^{-/-}$ and WT mice represented in **e**. (**d**) Cytokine array developed by pooling 3 serum replicates taken from *Prf1*$^{-/-}$ and WT female mice at the age of 2, 12, and 24 months. (**e**) Original photos of representative spleens shown in **Fig. 2g**. (**f**) Flow-cytometric quantification of infiltrating immune cells in the liver, pancreas, and lung of *Prf1*$^{-/-}$ female mice, compared to the WT female mice at the age of 2 months. The different immune subsets are presented as percent of total CD45 cells. Values are means ± SD, $n \geq 4$. (* $P < 0.05$).

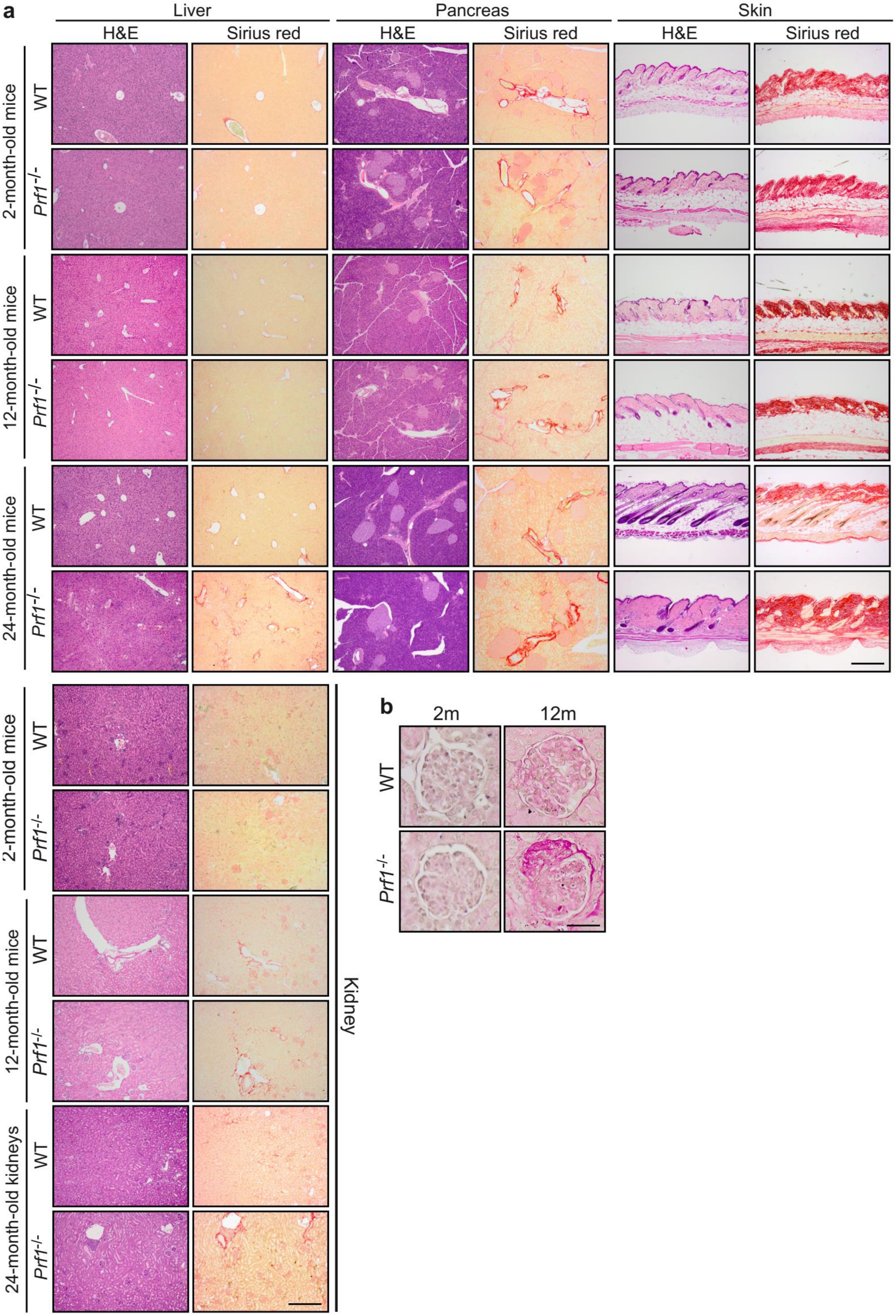
a
Liver
Pancreas
Skin
H&E
Sirius red
2-month-old mice
12-month-old mice
24-month-old mice
24-month-old kidneys
WT
Prf1-/-
Kidney
b
2m
12m

Supplementary Figure 4 *Prf1*$^{-/-}$ mice exhibit compromised organ function and impaired tissue integrity. (**a**) Representative images of H&E-stained and Sirius Red-stained sections of liver, pancreas, skin, and kidney from 2, 12, and 24 months old *Prf1*$^{-/-}$ and WT mice. The corresponding H&E-stained sections of skin from old mice are presented in **Fig. 3d**. Scale bar, 200 µm. (**b**) Representative images of PAS staining of glomeruli from kidneys of 2, and 12 months old *Prf1*$^{-/-}$ and WT mice. Scale bar, 25 µm.

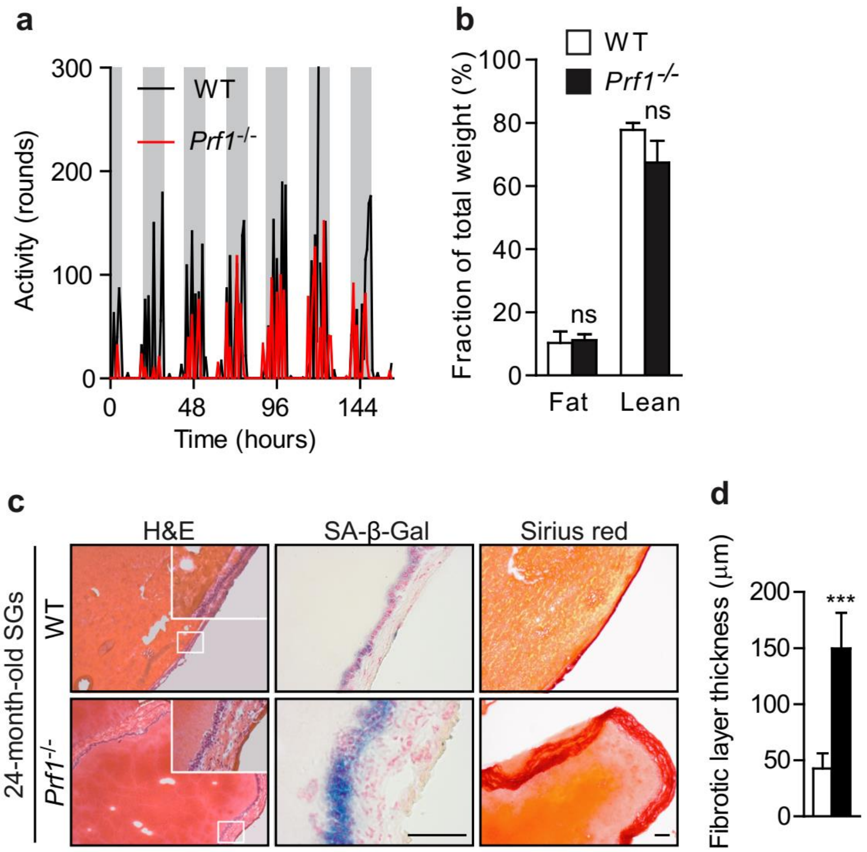

Supplementary Figure 5 *Prf1*$^{-/-}$ mice exhibit reduced fitness and early onset of age-related pathologies. (**a**) Histogram depicts the results of old *Prf1*$^{-/-}$ and WT male mice on a 7-day voluntary exercise test, measured by a running wheel. (**b**) Body composition of old *Prf1*$^{-/-}$ and WT male mice as was determined by EchoMRI. (**c**) Representative images of H&E, SA-β-Gal, and Sirius Red staining of seminal-gland sections from old *Prf1*$^{-/-}$ and WT mice. Scale bar, 100 µm. (**d**) Thickness of the fibrotic layer in the seminal glands of old *Prf1*$^{-/-}$ and WT mice, based on Sirius Red staining. Values are means ± SD; Student's t-test was used for all comparisons between *Prf1*$^{-/-}$ mice and WT mice. (*** $P < 0.001$).

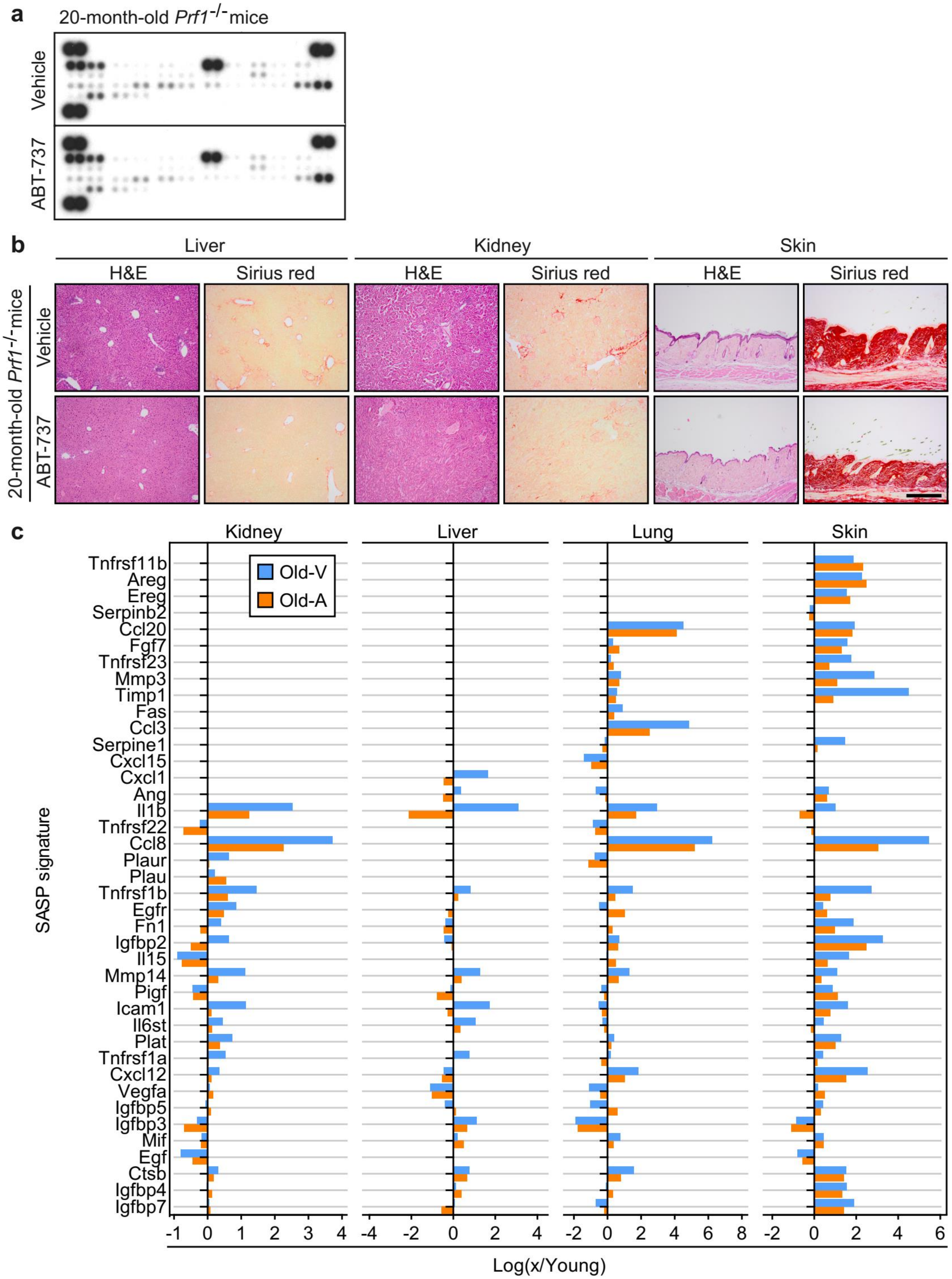
a
20-month-old Prf1-/- mice
Vehicle
ABT-737
b
Liver
Kidney
Skin
H&E
Sirius red
20-month-old Prf1-/- mice
Vehicle
ABT-737
c
Kidney
Liver
Lung
Skin
Old-V
Old-A
SASP signature
Tnfrsf11b
Areg
Ereg
Serpinb2
Ccl20
Fgf7
Tnfrsf23
Mmp3
Timp1
Fas
Ccl3
Serpine1
Cxcl15
Cxcl1
Ang
Il1b
Tnfrsf22
Ccl8
Plaur
Plau
Tnfrsf1b
Egfr
Fn1
Igfbp2
Il15
Mmp14
Pigf
Icam1
Il6st
Plat
Tnfrsf1a
Cxcl12
Vegfa
Igfbp5
Igfbp3
Mif
Egf
Ctsb
Igfbp4
Igfbp7
Log(x/Young)

Supplementary Figure 6 Treatment with ABT-737 counteracts accelerated aging process of *Prf1*$^{-/-}$ male mice. (**a**) Cytokine array developed by pooling 3 serum replicates taken from ABT-737-treated and vehicle-treated *Prf1*$^{-/-}$ mice at the age of 20 months. (**b**) Representative images of H&E-stained and Sirius Red-stained sections of liver, pancreas, skin, and kidney from ABT-737-treated and vehicle-treated *Prf1*$^{-/-}$ mice at the age of 20 months. Scale bar, 200 µm. (**c**) Log ratio of SASP genes found to be expressed in the tissues of 20-month old, ABT-737-treated and vehicle-treated *Prf1*$^{-/-}$, versus 3-month old *Prf1*$^{-/-}$ mice.

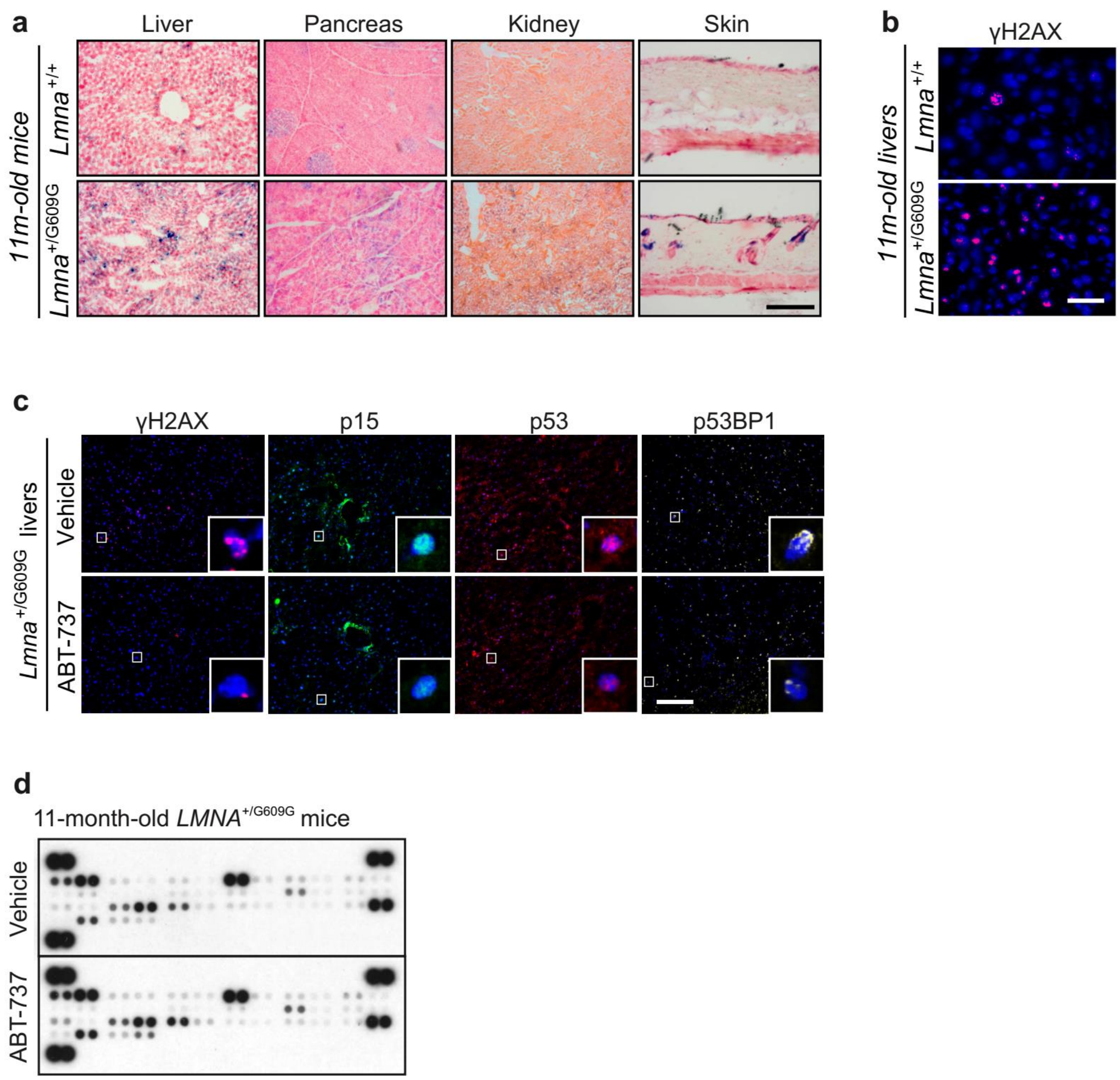

Supplementary Figure 7 Treatment with ABT-737 increases median lifespan of progeroid mice. (**a**) Representative images depicting SA-β-Gal activity, analyzed at 11 months, in frozen sections of livers, pancreas, kidney, and skin from *LMNA*$^{+/G609G}$ mice and control WT mice. Scale bar, 100 µm. (**b**) Representative images depicting immunofluorescence staining of γH2AX in livers from *LMNA*$^{+/G609G}$ mice and control WT mice. Scale bar, 100 µm. (**c**) Representative images depicting immunofluorescence staining of γH2AX, p15, p53, and p53BP1 in livers from ABT-737-treated and vehicle-treated *LMNA*$^{+/G609G}$ mice. Scale bar, 100 µm. (**d**) Cytokine array developed by pooling 3 serum replicates taken from ABT-737-treated and vehicle-treated *LMNA*$^{+/G609G}$ mice at the age of 11 months.

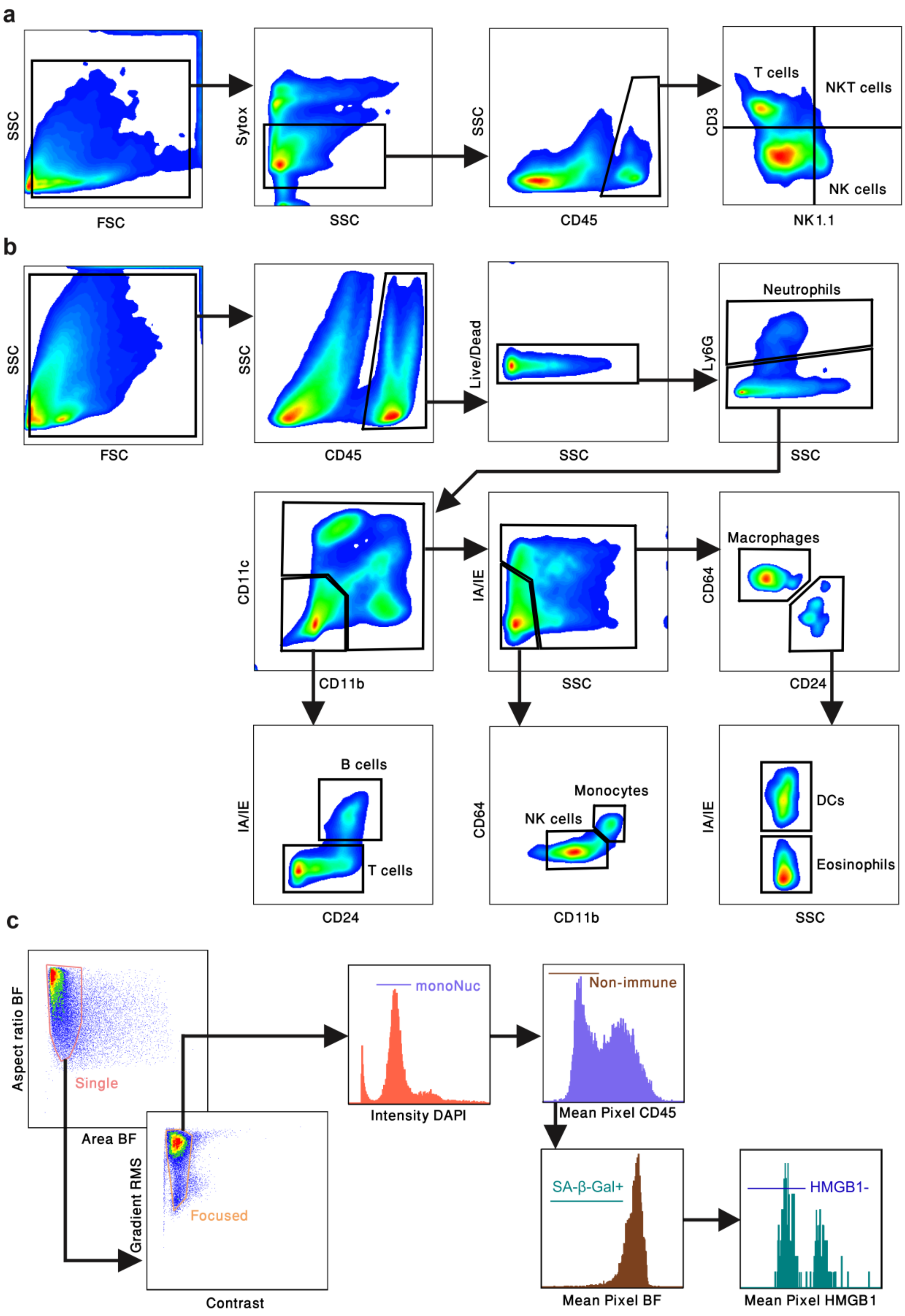

a
SSC
FSC
Sytox
SSC
SSC
CD45
CD3
NK1.1
T cells
NKT cells
NK cells
b
SSC
FSC
SSC
CD45
Live/Dead
SSC
Ly6G
SSC
Neutrophils
CD11c
CD11b
IA/IE
SSC
CD64
CD24
Macrophages
IA/IE
CD24
B cells
T cells
CD64
CD11b
Monocytes
NK cells
IA/IE
SSC
DCs
Eosinophils
c
Aspect ratio BF
Area BF
Single
Gradient RMS
Contrast
Focused
monoNuc
Intensity DAPI
Non-immune
Mean Pixel CD45
SA-β-Gal+
Mean Pixel BF
HMGB1-
Mean Pixel HMGB1

Supplementary Figure 8 Analysis of flow-cytometry and Imagestream X data. (**a**) Contour plots and gating strategy used for identification of tissue-reside cytotoxic cells by FlowJo v10 software as described in Figure 2b and Figure 4i. (**b**) Contour plots and gating strategy used for identification of infiltrating immune cells by FlowJo v10 software as described in Supplementary Figure 3f. (**c**) Contour plots and gating strategy used for identification of senescent cells in tissues by IDEAS 6.1 software as described in Figure 1i, Figure 4e, and Supplementary Figure 2b,c.

Supplementary references

1. Yen-Rei, A.Y.*, et al.* A protocol for the comprehensive flow cytometric analysis of immune cells in normal and inflamed murine non-lymphoid tissues. *PloS one* **11**, e0150606 (2016).
2. Robinson, M.D. & Smyth, G.K. Moderated statistical tests for assessing differences in tag abundance. *Bioinformatics* **23**, 2881-2887 (2007).
3. Robinson, M.D., McCarthy, D.J. & Smyth, G.K. edgeR: a Bioconductor package for differential expression analysis of digital gene expression data. *Bioinformatics* **26**, 139-140 (2010).
4. Robinson, M.D. & Oshlack, A. A scaling normalization method for differential expression analysis of RNA-seq data. *Genome biology* **11**, R25 (2010).
5. Yu, G.*, et al.* GOSemSim: an R package for measuring semantic similarity among GO terms and gene products. *Bioinformatics* **26**, 976-978 (2010).
