# Supplementary material for: Impaired immune surveillance accelerates accumulation of senescent cells and aging
Source: Nat Commun. 2018 Dec 21;9:5435. doi: 10.1038/s41467-018-07825-3 (PMC6303397; doi:10.1038/s41467-018-07825-3)
Supplement: Supplementary file 3 — Description of Additional Supplementary Files [file 41467_2018_7825_MOESM3_ESM.pdf]

Description of Additional Supplementary Files

File Name: Supplementary Data 1
Description: Differential expression.

File Name: Supplementary Data 2
Description: Scatterplot all genes.
